# Silencing of TGF-β1 in tumor cells impacts MMP-9 in tumor microenvironment

**DOI:** 10.1038/s41598-017-09062-y

**Published:** 2017-08-17

**Authors:** Lakisha D. Moore-Smith, Tatyana Isayeva, Joo Hyoung Lee, Andra Frost, Selvarangan Ponnazhagan

**Affiliations:** Department of Pathology, The University of Alabama at Birmingham, Birmingham, AL 35294 USA

## Abstract

Transforming growth factor (TGF)-β1 contributes to autocrine and paracrine functions in the tumor microenvironment (TME). The present study examined the effects of TGF-β1 crosstalk in TME and its role in mediating tumor formation and progression by targeted abrogation of TGF-β1 expression in metastatic cells *in situ*. Using species-specific primers, we found a significant increase in MMP-9 gene expression in the tumor-reactive stroma during late-stage metastasis in the lung. This effect was also confirmed in cancer-associated fibroblasts (CAFs) when co-cultured with the tumor cells. Knockdown of TGF-β1 expression in the tumor cells negatively affected matrix metalloproteinase (MMP)-9 gene expression. Fibroblasts, cultured in the presence of tumor cells with intact TGF-β1, showed a significant increase in proliferation rate, as well as expression of VEGF, bFGF, and SDF-1, which was not seen when TGF-β1 expression was abrogated in tumor cells. Absence of TGF-β1 in tumor cells also failed to result in myofibroblast differentiation. Co-implantation of CAFs and tumor cells with either intact TGF-β1 expression or devoid of TGF-β1 *in vivo* showed a significant increase in tumor growth kinetics in both cell types, suggesting a possible activation TGF-β receptor signaling in tumor cells in response to TGF-β from the TME.

## Introduction

The concept of “seed and soil” suggests that by exerting regulatory functions and selective pressures, the tumor microenvironment (TME) determines and shapes the malignant phenotype of cancer cells and promotes metastasis^1^. Thus, the TME has become an important area of study and a potential therapeutic target over the past decade. Previous studies suggested that tumor cells utilize autocrine and paracrine signaling pathways to promote growth and metastasis, independent of the microenvironment^2–4^. However, it is now apparent that tumors are composed of heterogenous tumor cells, endothelial cells, immune cells, smooth muscle cells, and fibroblasts. It has been shown that cross-talk between these cells helps to promote metastasis^5–8^. Tumor cells secrete several factors, which modify the surrounding stroma. Fibroblasts in the TME are greatly modified by soluble factors produced by the tumor cells, thereby forming a tumor-reactive stroma, and may undergo differentiation into myofibroblasts and secrete additional growth factors, cytokines and chemokines, including: vascular endothelial growth factor (VEGF), fibroblast growth factor (FGF) and stromal-derived factor (SDF)-1, which act in both paracrine and autocrine manner^7–11^. Studies have shown that TGF-β signaling is an important factor in the cross-talk between tumor and stromal cells^5, 6^. Overexpression of TGF- β is a hallmark of several cancers and elevated TGF-β by tumor cells has been associated with various protumorigenic events^12–15^. Secretion of TGF-β from the tumor cells can act in a paracrine fashion to stimulate differentiation and proliferation of fibroblasts and endothelial cells. The reactive stromal cells in TME can mediate an invasive phenotype through the up-regulation VEGF, basic FGF, SDF-1 and matrix metalloproteinases (MMPs), which signal to the tumor cells to acquire a more migratory phenotype^7–11^. Up-regulation of TGF-β has also been shown to induce differentiation of fibroblasts to myofibroblast phenotype^9, 10, 16^.

Matrix metalloproteinases, a family of proteases secreted from the myofibroblasts and tumor cells, are important for remodeling of the matrix and aid in the migration and invasion of the tumor cells^17–19^. Transforming growth factor-beta (TGF-β) is a pleiotropic cytokine that plays a crucial function in a variety of malignancies^20, 21^. TGF-β is secreted as a latent protein, which is activated through cleavage of the inactive portions. The active portion of TGF-β is a 25 kDa homodimeric peptide that binds to TGF-β receptors and causes activation of downstream intracellular signaling molecules^22, 23^. Many epithelial cancer cells have been shown to up-regulate the expression of TGF-β to mediate pro-metastatic effects, while selectively ignoring the growth inhibitory effects^24^. This dual role of TGF-β signaling in modulating different mechanisms of tumor progression makes it an interesting therapeutic target for the treatment of localized and metastatic disease. Thus, targeting TGF-β ligand and receptor have indicated varying outcome on tumor growth in preclinical animal models and in human clinical trials^25, 26^.

Previous studies from our laboratory and others have shown that TGF-β1 acts through autocrine signaling to promote epithelial-mesenchymal transition in the tumor cells, and increase migration and invasion^27–29^. However, in addition to its effects on the tumor cell, TGF-β has been shown to stimulate angiogenesis through VEGF and bFGF expression, immunosuppression and expression of other growth factors^22^. Studies have shown that increased TGF-β expression in tumor cells correlates with increased vessel density in the tumor mass. Identification of the pleiotropic effects of TGF-β has resulted in a few targeted therapies mainly using antibodies and small molecule inhibitors, targeting either the ligand or TGF-β receptors^25, 26^. Unfortunately, results of the clinical trials so far, targeting TGF-β pathway, have not been successful, which indicates a need to understand more on the role of TME in promoting TGF-β signaling.

In order to further understand the effects of tumor-derived TGF-β1 in cells of the TME, the present study first identified the effects of TGF-β1 gene expression in major protumorigenic factors in the TME, both in the primary tumor and at a metastatic site in a transplantable tumor model in mice. Information derived from the *in vivo* studies was then used to characterize the effects of TGF-β1, specifically in normal-, and cancer-associated fibroblasts in a series of co-culture studies. Finally, the influence of tumor-reactive fibroblasts on tumor growth *in vivo* was determined in the presence or absence of TGF-β1 expression in tumor cells. Results of our studies indicated that silencing of TGF-β1 in tumor cells *in situ* exerted a significant effect on tumor fibroblast proliferation, differentiation and expression of protumorigenic growth factors, in particular, MMP9, as well as myofibroblast differentiation. Absence of TGF- β1 within tumor cells significantly decreased the growth of tumors *in vivo*, but co-transplantation of CAFs with tumor cells lacking TGF-β1 resulted in a significant increase in tumor growth. These results indicate that development of TGF-β targeted therapies delivered to both tumor cells and cells in TME may offer better scope for minimizing the rate of organ metastasis.

## Results

### Influence of tumor-associated fibroblasts on growth characteristics of MDA-MB-435 cells

**Figure 1:**
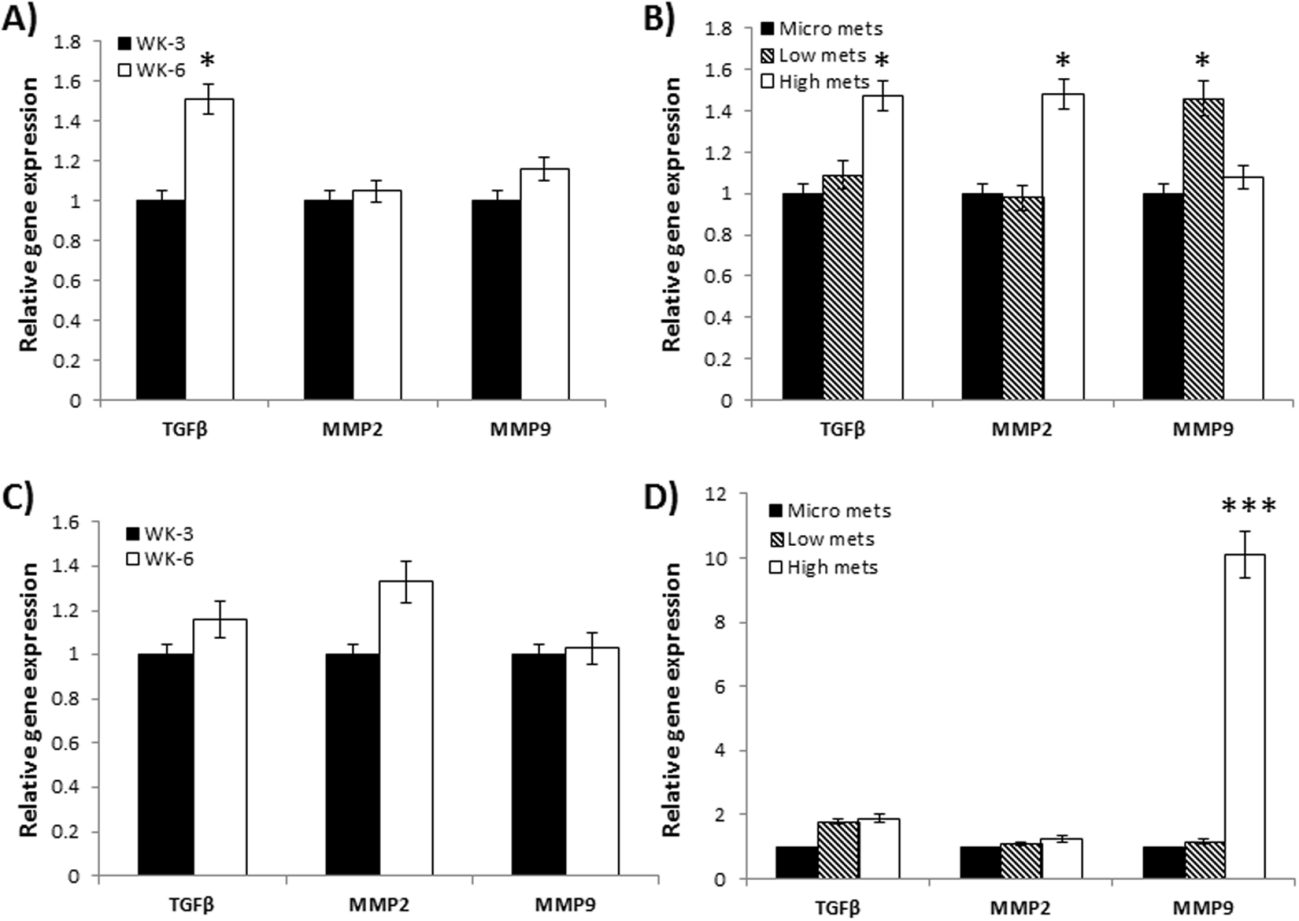
*In vivo* analysis of tumor versus stromal gene expression at various stages of tumor progression. MDA-MB-435 cells were subcutaneously injected into 8 week-old athymic nude mice. Primary tumors and lung metastases were allowed to develop over a 12 week period. From cohorts of mice, both primary tumor material and metastatic lesions in the lung were collected and RNA was isolated for analysis of TGF-β1, MMP2 and MMP9 expression in the tumor and stromal compartments. Species-specific primers were used to analyze gene expression in different compartments. Analysis of tumor (human) gene expression at the primary site on week-3 and on week-6 (**A**) and in the lung metastasis (**B**), as determined by QRT-PCR is shown. Those specific for stromal gene (mouse) expression at the primary site and in lung metastasis are shown in **(C)** and **(D)**, respectively. *p < 0.05; ***p < 0.001.

In order to establish the significance of tumor-reactive stroma on tumor growth, and to determine if this influence is impacted by TGF-β1 *in vivo*, we first analyzed the expression of key molecules in both tumor and stromal compartments using species-specific primers. MDA-MB-435 cells were implanted subcutaneously in athymic nude mice and the primary tumors were explanted when the tumor volume reached 100 mm^3^ around week 6 to facilitate metastasis^30^. From additional cohorts of mice, primary tumors were also excised at three weeks and six weeks post tumor cell implantation and RNA was collected for analysis. Upon sacrifice, mice were divided into three groups based on size and amount of lung metastasis: 1) mice with microscopic lesions, 2) low copy (<10) macroscopic lesions and 3) high (>10) macroscopic lesions. The lungs were harvested and RNA collected from the tissue for gene expression analysis. Using primers specific for human gene transcripts, the relative gene expression was determined in the tumor, and by using mouse-specific primers, contribution from the stromal cells was analyzed for TGF-β1, MMP-9 and MMP-2. Results, shown in Fig. 1A, indicated that only expression of TGF-β1 was significantly increased in week-6 of primary tumor. However, the expression levels of MMP-2 and MMP-9 did not change significantly in primary tumor. However, significant increase in TGF-β1, MMP-2 and MMP-9 gene expression was noted in tumor cells from high metastatic lesions in the lungs (Fig. 1B). Analysis of TGF-β1, MMP-9 and MMP-2 transcripts in stromal compartment using mouse-specific primers did not indicate a significant increase in TGF-β1, MMP-2 or MMP-9 expression at the primary site (Fig. 1C), but there was a highly significant increase in MMP-9 gene expression in stromal compartment in lungs with high metastasis (Fig. 1D). These data prompted that regulation of MMP-9 expression in the stromal cells may play an important role in tumor-stroma cross talk during tumor progression.

### Effects of tumor-derived TGF-β1 on cell proliferation through paracrine signaling

**Figure 2:**
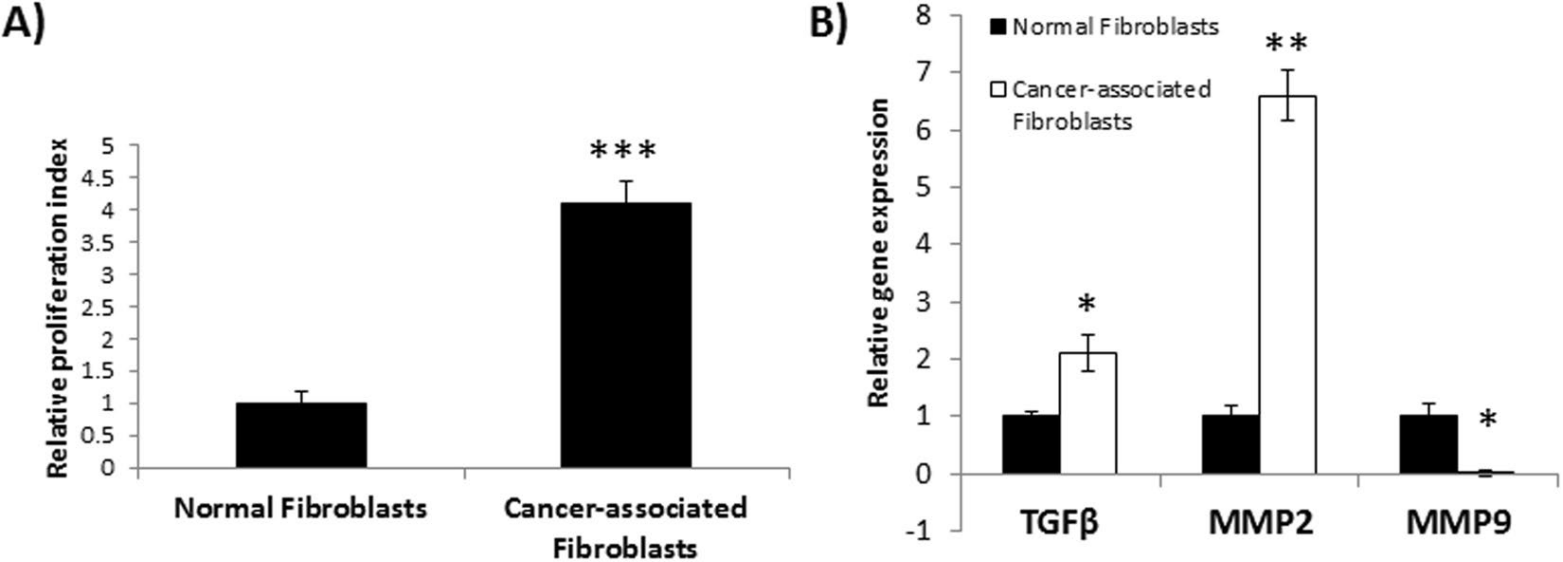
Characterization of normal fibroblasts and cancer-associated fibroblasts *in vitro*. Primary fibroblast cultures from patients with normal mammary epithelial and cancerous epithelium were obtained and analyzed for differences in proliferation and TGF-β1, MMP2 and MMP9 gene expression. An MTT proliferation assay was used to determine changes in proliferation in tumor associated fibroblasts **(A)**. RNA was isolated from replicate cultures and analyzed for TGF-β1, MMP-9 and MMP-2 gene expression using RT-PCR **(B**). *p < 0.05; **p < 0.01; ***p < 0.001.

**Figure 3:**
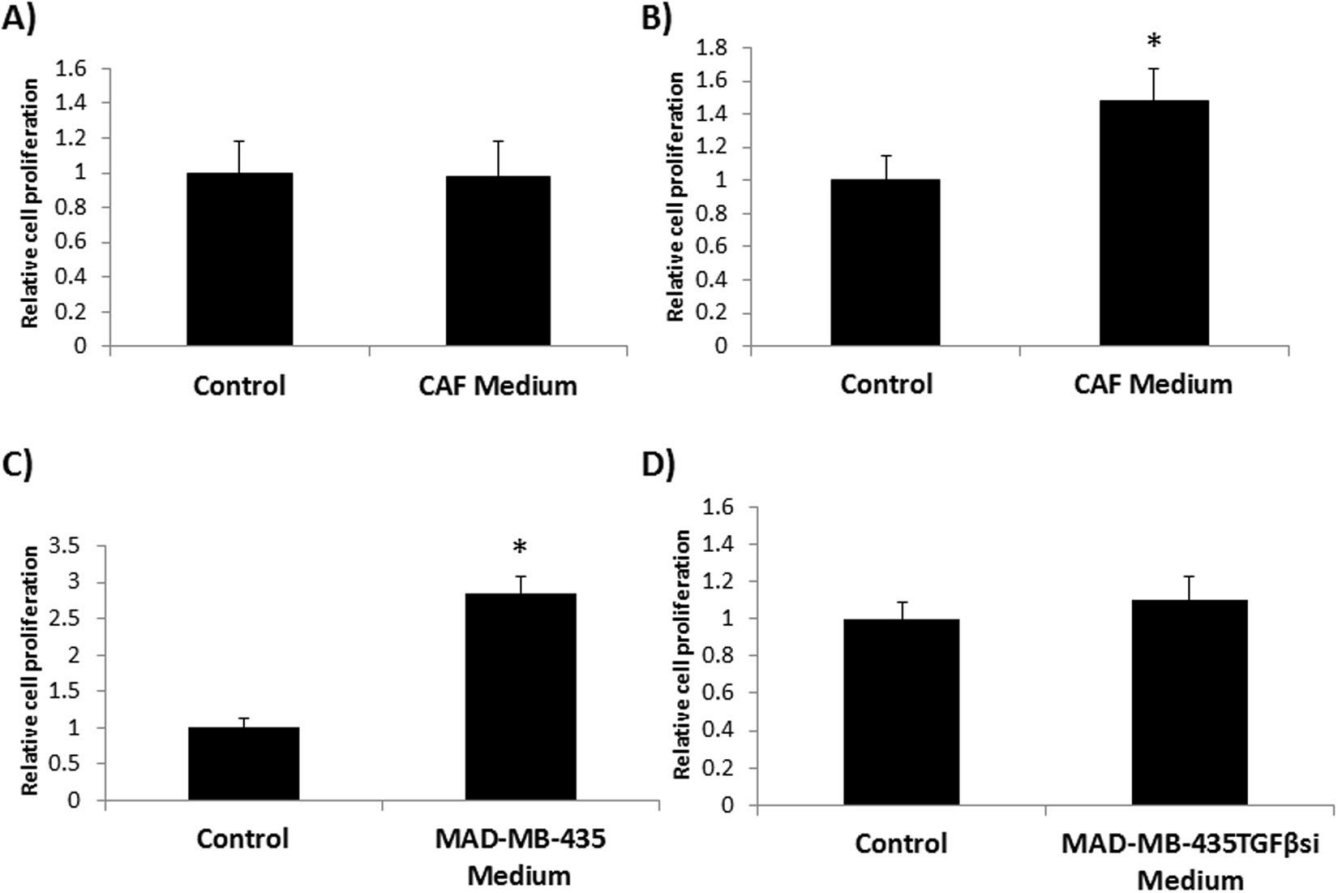
Co-culture assay to determine effects of paracrine signaling from tumor cells and CAFs. MDA-MB-435 **(A**) and MDA-MB-435TGFβsi **(B)** cells were cultured in CAF conditioned-medium for 72 hrs and then analyzed for proliferation using MTT assay. Similarly, CAFs were also cultured in conditioned medium from either MDA-MB-435 **(C)** or MDA-MB-435TGFβsi **(D)** cells for 72 hrs and changes were determined by MTT assay. *p < 0.05.

Several studies have shown that both autocrine and paracrine signaling play a significant role in mediating tumorigenesis. Growth factors secreted by the tumor and stromal cells may have an influence on several cellular properties including proliferation^30^. Based on the results of *in vivo* studies, next, we determined the paracrine effects of tumor-derived soluble factors on growth and proliferation of normal fibroblasts (NFs) and cancer-associated fibroblasts (CAFs). First, we compared the kinetics of proliferation, and gene expression levels of TGF-β1, MMP-2 and MMP-9 between NFs and CAFs. Results of this study indicated a significant increase in proliferation of CAFs (Fig. 2A) and in the expression of TGF-β1 and MMP-2 (Fig. 2B). Interestingly, MMP-9 level in CAFs was significantly lower than that in NFs (Fig. 2B). Based on our observation that MMP-9 was significantly elevated at late stage metastasis in tumor cells *in vivo*, we focused in particular on tumor- derived TGF- β1 in regulating MMP-9 expression. Using conditioned media from both tumor cells and CAFs, we determined the effects of secreted factors on proliferation of MDA-MB-435 cells with intact TGF-β1 expression or clonal derivatives of the parental MDA-MB-435 cell line, abrogated in TGF-β1 expression using stable shRNA transfection (MDA-MB-435TGFβsi; 31). Results of this study indicated that stimulation of MDA-MB-435 cells with CAF conditioned medium had no effect on the proliferation rate of tumor cells (Fig. 3A), but there was a significant increase in the proliferation of MDA-MB-435TGFβsi cells upon treatment with conditioned-medium from CAFs (Fig. 3B). Further, stimulation of CAFs with conditioned media from MDA-MB-435 cells resulted in a significant increase in the proliferation (Fig. 3C), but not conditioned medium from or MDA-MB-435TGFβsi cells (Fig. 3D), signifying a role for TGF-β1 in stimulating proliferation of fibroblasts in the TME.

### Analysis of gene expression in tumor-stroma co-culture systems

**Figure 4:**
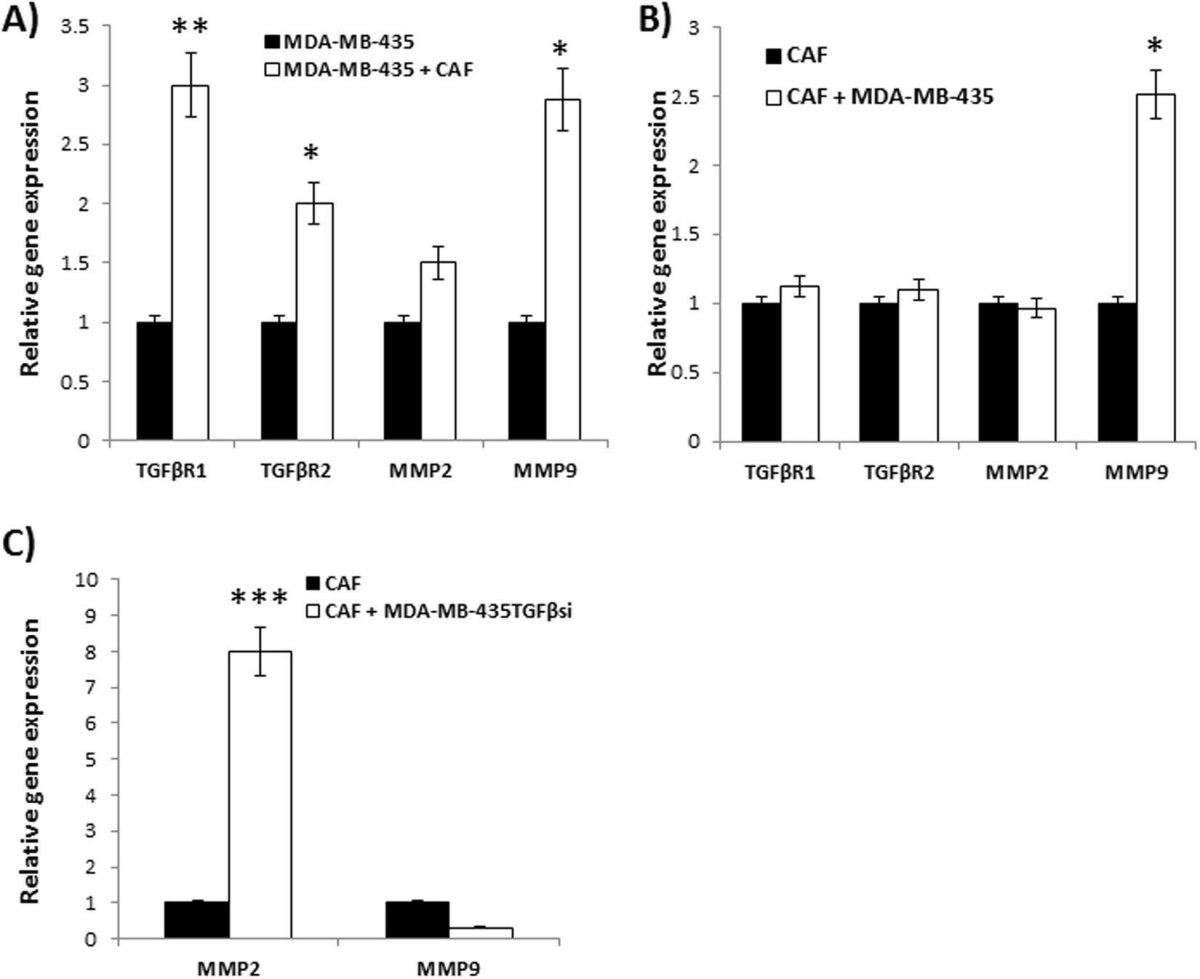
Co-culture assays to determine the effects of paracrine signaling on tumor and stromal cell gene expression. MDA-MB-435 cells and MDA-MB-435 TGF-βsi cells were co-cultured with fibroblasts in Boyden chambers. Forty-eight hrs later, RNA was collected from both the tumor and fibroblasts for RT-PCR analysis of TGF-β1, TGF-β2, TGF-β3, TGFβRI, TGFβRII, MMP-2 and MMP-9 gene expression levels. Values provided in (**A**) indicate gene expression from MDA-MB-435 cells, grown with CAFs; (**B**) in CAFs, grown with MDA-MB-435 cells; (**C**) in CAFs, grown with MDA-MB-435TGFβsi cells. *p < 0.05; **p < 0.01; ***p < 0.001.

Next, we determined the effects of TGF-β1 on expression of TGF-β receptors 1 and 2, MMP-2, and MMP-9 in fibroblasts using a co-culture assay. MDA-MB-435 and MDA-MB-435TGFβsi cells were co-cultured in Boyden chambers with fibroblasts. Cells were plated in a 3:1 ratio of tumor cells to fibroblasts and allowed to grow for 72 hrs prior to gene expression analysis. In co-cultures with MDA-MB-435 and fibroblasts, the tumor cells showed a significant increase in TGF-β type 1 and type 2 receptors and MMP-9 expression (Fig. 4A). Analysis of expression of the same proteins in fibroblasts, co-cultured with MDA-MB-435 cells, showed no significant increase in TGF-βR1, R2 or MMP-2 expression, when compared to cells cultured alone. However, in contradistinction, MMP-9 expression showed a 3-fold increase (Fig. 4B). This result corresponds to the *in vivo* data in which MMP-9 expression was increased in the tumor-reactive stroma as the tumor progressed. When fibroblasts were co-cultured with MDA-MB-435TGFβsi cells, it resulted in a significant decrease in MMP-9 gene expression in them (Fig. 4C). Interestingly, there was a 10-fold increase in MMP-2 gene expression in the fibroblasts when co-cultured with MDA-MB-435TGFβsi cells (Fig. 4C).

### Effects of tumor-derived TGF-β1 on proliferation of tumor-reactive stroma and epithelial- mesenchymal transition

**Figure 5:**
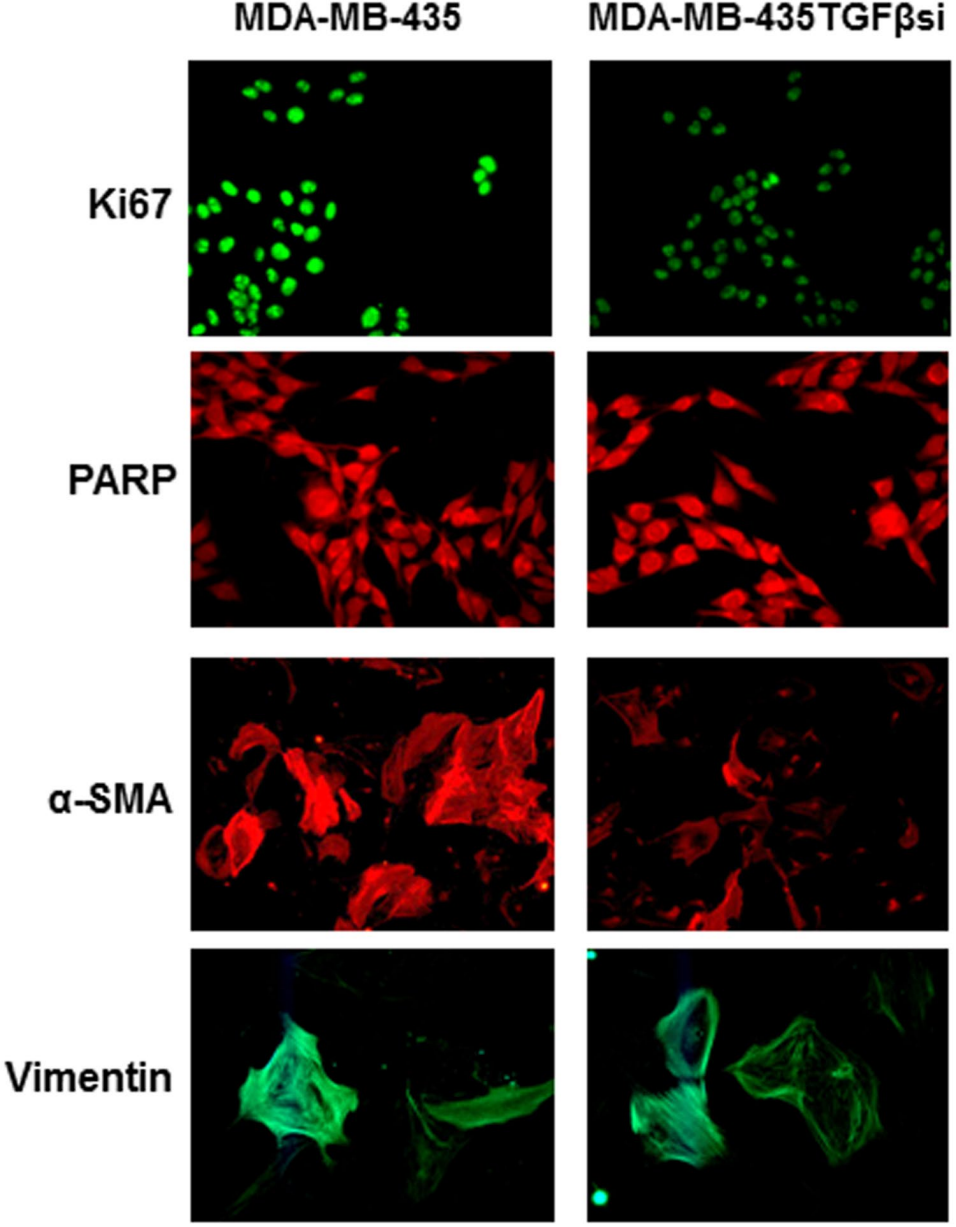
Co-culture assays to determine the effects of paracrine signaling on stromal cell proliferation, apoptosis and fibroblast differentiation. Fibroblasts were cultured in conditioned medium from MDA-MB-435 or MDA-MB-435-TGFβsi cells for 72 hrs. From replicate cultures, cell proliferation was determined using Ki67 staining and apoptosis determined using PARP-p85 staining. To determine myofibroblast differentiation cells were stained with alpha-smooth muscle actin (α-SMA). Vimentin staining was performed as a control for the mesenchymal cell lineage. Images were captured using a Leica fluorescence microscope (x20).

A hallmark of phenotypic changes of epithelial cancers in advanced stages includes mesenchymal transition. Hence, we next sought to determine if TGF-β1, produced by cancer cells, particularly affects epithelial cell morphology in the vicinity. To this end, CAFs were cultured in the presence of conditioned medium from either MDA-MB-435 or MDA-MB-435-TGFβsi cells for 48 hrs and then fixed for staining by immunocytochemistry. Ki67 staining was used to determine the changes in proliferation of the CAFs. CAFs incubated with MDA-MB-435 cell conditioned medium showed an increase in Ki67 staining when compared to cells cultured in MDA-MB-435-TGFβsi medium (Fig. 5). PARP p85 subunit staining was used as a marker for apoptosis. Results indicated no significant difference in PARP staining between CAFs, cultured in MDA-MB-435 or MDA-MB-435TGFβsi conditioned media. To determine whether tumor-derived TGF-β enhances transdifferentiation of stromal epithelium into myofibroblasts, alpha-smooth muscle actin (α-SMA) was used, and to confirm mesenchymal lineage, vimentin staining was used. Results indicated an increase in α-SMA staining when CAFs were cultured in MDA-MB-435 conditioned medium compared to cells cultured with MDA-MB-435-TGFβsi conditioned medium (Fig. 5). However, there was no such difference in vimentin expression when CAFs were treated with medium from MDA-MB-435 or MDA-MB-435TGFβsi cells.

### Analysis of major growth factors and chemokine expression in CAFs

**Figure 6:**
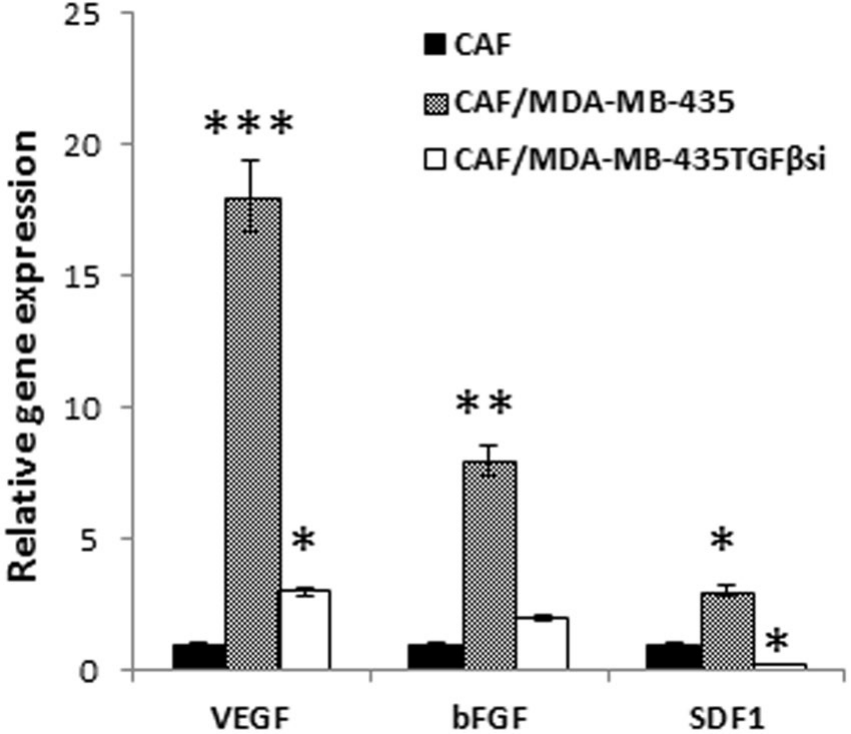
Analysis of growth factor expression in co-cultured fibroblasts. CAFs were cultured in Boyden chambers with either MDA-MB-435 cells or MDA-MB-435TGFβsi cells for 72 hrs. RNA was collected from the cells and analyzed for VEGF, bFGF and SDF-1 expression. RT-PCR analysis was performed in triplicates and normalized to GAPDH values and expressed as relative gene expression to gene expression levels in control fibroblasts. *p < 0.05; **p < 0.01; ***p < 0.001.

Recent studies have shown that cancer-associated fibroblasts, when activated as tumor-reactive stroma, increase expression of growth factors that promote tumor growth and metastasis. To discern the significance of tumor-derived TGF-β1 in particular on this effect, we analyzed the expression of key growth factors VEGF, bFGF and chemokine SDF-1 in fibroblasts that were co-cultured with either MDA-MB-435 or MDA-MB-435TGFβsi cells. The cells were cultured in Boyden chambers for 72 hrs and then RNA was isolated and subjected to qRT-PCR to determine gene expression. As shown in Fig. 6, CAFs, cultured with MDA-MB-435 cells, illustrated a significant increase in VEGF, bFGF and SDF-1 gene expression. When CAFs were cultured with MDA-MB-435TGFβsi cells, there was a modest, yet, significant increase in VEGF, but there was no significant increase in bFGF gene expression. Interestingly, expression of SDF-1 decreased dramatically by more than 6-fold (Fig. 6).

### Analysis of primary tumor growth *in vivo*

**Figure 7:**
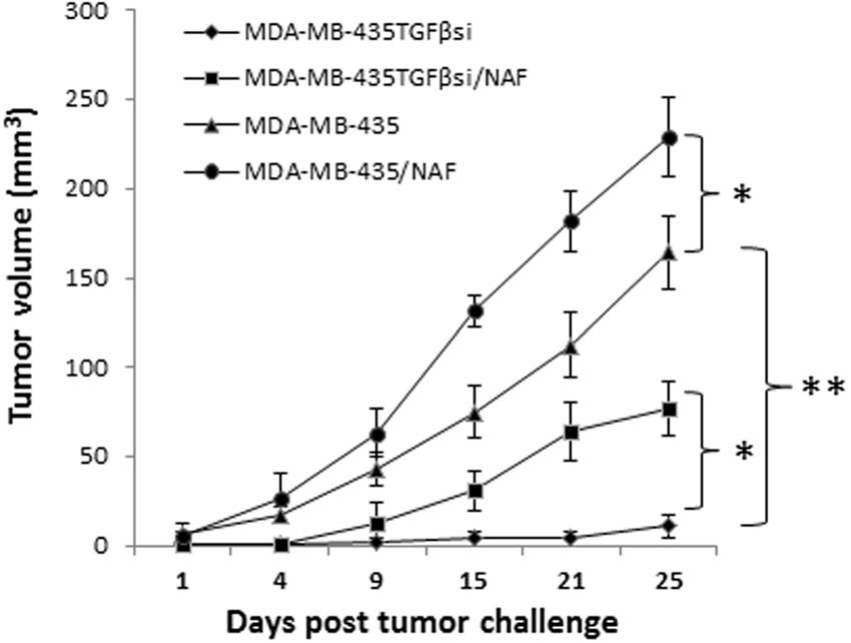
Effects of CAFs on the *in vivo* growth of primary tumors with intact or abrogated in TGFβ1 expression. Athymic nude mice were injected subcutaneously with MDA-MB-435 cells or MDA-MB-435TGFβsi cells either alone or admixed with CAFs. The cells were allowed to grow for 25 days and tumor volumes were measured every two days to determine growth kinetics. *p < 0.05; **p < 0.01.

Finally, an *in vivo* study was performed to determine the influence of tumor-derived TGF-β1 in tumor-stroma interactions on primary tumor growth. MDA-MB-435 and MDA-MB-435TGFβsi cells, either alone or in combination with CAFs, were implanted subcutaneously in nude mice, and tumor growth was measured every other day. Results, shown in Fig. 7, not only indicated that primary tumor growth between different groups was significantly different, but also demonstrated a significant influence of CAFs in promoting tumor growth, even in the absence of TGF-β1 in tumor cells *in situ*. There was a significant difference in mean tumor volume between MDA-MB-435 and MDA-MB-435TGFβsi tumors, when transplanted alone (Fig. 7; P < 0.05). When the cells were co-implanted with CAFs, there was a significant increase in tumor growth kinetics in both tumor types. The difference was more than 6-fold (85%) in the growth of MDA-MB-435TGFβsi tumors with CAFs compared to the parental MDA-MB-435 cells, implanted with CAFs (39%).

## Discussion

Although most anti-cancer therapies are designed to target tumor cells, a better understanding of tumor heterogeneity and signaling from the TME is rapidly evolving, suggesting tumor cells may not be the only sufficient target when developing cancer therapeutics. New data suggest that tumor cells either create or are drawn to a permissible microenvironment which is very different from that seen in normal tissue^31, 32^. Studies have shown that remodeling of the microenvironment is an important process that stimulates growth of the primary tumor and homing of metastatic cells to secondary sites^33, 34^. Alterations such as vascular remodeling, fibroblast differentiation and degradation of the basement membrane are some of the events that must occur to promote tumorigenesis; eventually leading to metastasis^22^. These processes, in turn, also give rise to a reactive-stroma, which acts synergistically to promote growth and invasion of tumor cells.

Signaling between tumor cells and fibroblasts is one of the main events that contributes to the production of a reactive stroma. When tumor cells secrete factors such as TGF-β, fibroblasts respond by up-regulating MMP, VEGF, bFGF, and SDF-1, all of which have been shown to play a major role in mediating tumorigenesis^35, 36^. TGF-β signaling has also been shown to be associated with differentiation of fibroblasts into myofibroblasts^37^. Such differentiation may be important because myofibroblasts express a different subset of cytokines and growth factors that contribute to tumor progression^37^. Studies have also shown that in a “humanized” mouse mammary gland, injections of tumor cells with CAFs increased the tumor progression when compared to cells injected with normal fibroblasts^38^. Results of studies from our *in vivo* model using species-specific primers allowed us to differentially analyze human gene expression from the implanted tumor cells and stromal gene expression from the mouse stroma. Our results indicated that over the progression of disease from primary tumor to lung metastasis, there was a significant increase in MMP-9 gene expression. The increase in MMP-9 expression was not seen in the tumor cells, but only in the stromal compartment.

From results of the present study, up-regulation of MMP-9 appears to be directly mediated by TGF-β1 signaling from tumor cells since abrogation of TGF-β1 within tumor cells led to a decrease in MMP-9 levels. MMP-9 is a member of a large family of matrix metalloproteinases. The specific action of this protease is breakdown of the extracellular matrix under both physiological and pathological conditions. Upregulation and membrane localization of MMP-9 is important for degradation of the basement membrane, specifically collagen type IV, which allows for intravasation of the tumor cells into circulation^18, 19^. However, MMP-9 can also mediate a positive feedback mechanism due to its ability to activate latent TGF-β. Once activated, TGF-β can signal surrounding fibroblasts to increase expression of MMP-9. When normal fibroblasts were compared with CAFs, expression of both TGF-β and MMP-2 was significantly up-regulated in the CAFs but MMP-9 levels were decreased. These data support the notion that TGF-β1 signaling, specifically from the tumor cells, acts to initiate expression of MMP-9. Interestingly, this effect was reversed when the fibroblasts were cultured with MDA-MB-435TGFβsi cells. However, there was an associated increase in MMP-2 expression suggesting that compensatory mechanisms may be involved in maintaining high levels of metalloproteinases in the TME. Whether TGF-β1 exerts a direct effect on MMP-9 in fibroblasts or an indirect effect through TGF-β/Smad signaling pathway needs to be elucidated.

Analysis of CAF differentiation using conditioned medium from tumor cells indicated that TGF-β1, secreted by the tumor cells, can stimulate differentiation of fibroblasts into myofibroblasts. Increased proliferation was seen in fibroblasts, cultured with MDA-MB-435 conditioned medium as measured by an MTT assay and by Ki67 staining. However, this effect was decreased when the fibroblasts were cultured with MDA-MB-435TGFβsi cells. Furthermore, alpha-smooth muscle actin (α-SMA) demonstrated an increase when fibroblasts were cultured with MDA-MB-435 cells, compared to co-culturing with tumor cells lacking TGF-β1 expression.

TGF-β1 has been extensively studied in the context of both angiogenesis and immune suppression. It has been shown in several studies that TGF-β mediates expression of VEGF, bFGF and SDF-1 in the TME to promote endothelial and fibroblast cell proliferation and migration. VEGF is a growth factor that has been well studied for its role in angiogenesis. Increased expression of VEGF from tumor and stromal cells can increase endothelial cell proliferation and migration. SDF-1 is expressed by stromal cells including fibroblasts and endothelial cells. This chemokine is up-regulated in the TME and promotes cell proliferation, migration and mediates metastasis and/or homing to the secondary site^19, 39^. Basic FGF is also a potent mediator of cell proliferation and angiogenesis in TME. These findings were also supported from our studies in which fibroblasts were cultured with conditioned media from the tumor cells and analyzed for growth factor expression. However, there was no increase in VEGF, bFGF, and SDF-1 gene expression when fibroblasts were cultured with MDA-MB-435TGFβsi cells, highlighting the pleiotropic significance of TGF-β1.

A reciprocal effect of CAFs on the growth of tumor cells was evident from the present study. Superimposing these data, it was interesting to note a significant increase not only in tumor growth kinetics when MDA-MB-435 cells were implanted with CAFs, but also in the growth kinetics of tumor cells abrogated in TGF-β1expression. This suggests inhibiting TGFβ in both the epithelial and stromal cells in TME is critical for therapies targeting TGF-β1, especially in late-stage disease where tumor-reactive stroma in the metastatic site would have acquired a protumorigenic transition, expressing elevated levels of TGF-β1. Collectively, it is evident from our results that TGF-β1 cross-talk between tumor cells and fibroblasts mediate key events in tumor progression, resulting in metastasis.

Recent studies have shown that fibroblasts, immune cells, and endothelial cells can secrete TGF-β into TME^5–8, 11^. Fibroblasts are the most abundant cell type in TME and provide support for the tumor growth at both primary and secondary sites. Our results suggest that inhibition of TGF-β1 signaling in the TME can decrease conversion of the stroma into a more reactive environment that supports tumorigenesis. Thus targeting TGF-β in both stroma and tumor cells *in situ* would improve TGF-β targeted therapies for cancer. Recent studies have confirmed melanoma origin of MDA-MB-435 cells, disproving the notion that they were of breast cancer origin as previously believed^40^. Notwithstanding this, overexpression of TGF- β in several human malignancies provides this pleiotropic growth factor as a possible target for cancer therapy. A better characterization and new methods of delivery should also help move TGF-β-targeted therapies to the forefront as possible approaches for treatment of metastatic disease. Results from this study can be extrapolated to many other epithelial cancers that are characterized by the influence of TGF-β signaling in tumor growth and metastasis.

## Materials and Methods

### Reagents, cell lines, and institutional approval

The metastatic human melanoma cell line MDA-MB-435 was a generous gift from Dr. Janet Price (MD Anderson Cancer Institute, Houston, TX). The cells were maintained in L-15 medium supplemented with 10% FBS, 1 mM sodium pyruvate, 2x non-essential amino acids, 2 mM L-glutamine and 100 μg/ml penicillin-streptomycin. Cells were grown in a 5% CO_2_ incubator at 37 °C. Primary fibroblasts were isolated from patients with epithelial cancer (CAF) and normal controls (NAF). Cells were maintained in DMEM containing low glucose (1 mg/ml), 10% FBS and 100 μg/ml penicillin-streptomycin. Molecular biology reagents were purchased from New England Biolabs Inc. (Beverly, MA). Monoclonal antibodies for ki67, α-SMA, PARP, and vimentin were purchased from Cell Signaling (Danvers, MA). All animal experiments were performed with approved protocol from UAB Institutional Animal Care and Use Committee (UAB-IACUC) and following the guidelines of UAB-IACUC for animal care and use.

### Cell proliferation assay

An MTT assay was used to determine cell proliferation, as published earlier^30^. Briefly, 5000 cells were plated per well in a 96-well plate and allowed to attach overnight. Cells were serum-starved overnight and then grown for 72 hrs prior to analysis. Normal fibroblasts or cancer-associated fibroblast, unmodified MDA-MB-435 cells or clonal derivatives of MDA-MB-435 cells lacking TGF-β1 expression (MDA-MB-435TGFβsi) through constitutive expression of siRNA^30^ were then incubated with 12 mM MTT stock solution for 4 hrs at 37 °C and then 100 µl of SDS-HCl solution was added to each well and incubated overnight. Absorbance was read at 570 nm in a plate reader.

### Quantitative real-time polymerase chain reaction

Quantitative PCR was performed using the SYBR Green detection system from Bio-Rad (Hercules, Ca.). Total RNA was isolated using TRIzol reagent (Invitrogen) as per manufacturer’s instructions and then reverse-transcribed into cDNA using Bio-Rad’s iScript cDNA synthesis kit. Quantitative real-time PCR was performed using Bio-Rad’s SYBR green supermix and iCycler detection system (Bio-Rad Hercules, CA.). The primer sequences used for different transcripts are listed in Table 1. PCR assays were performed in triplicate and values were normalized to GAPDH levels as internal control. Polymerase chain reactions were performed with a 3 min pre-incubation, at 95 °C, followed by 45 cycles of: 15 sec denaturation at 95 °C, 30 sec each for annealing and extension at 57 °C. PCR products were subjected to melting curve analysis using the light cycler system to exclude amplification of non-specific products. Quantitation of the PCR data was performed using the ΔCT method as described previously^30, 41^.

**Table 1:** Primers used in quantitiative real-time PCR.

Gene	Human	Mouse
TGF-β1	F.P. 5′-GAGGGGAAATTGAGGGCTTT-3′	F.P. 5′-CACCGGAGAGCCCTGGATA-3′
	R.P. 5′-CGGTAGTGAACCCGTTGATG-3′	R.P. 5′-TGTACAGCTGCCGCACACA-3′
MMP-2	F.P. 5′-GAAGGTGAAGGTCGGAGT-3′	F.P. 5′-CACACCAGGTGAACCATGTG-3′
	R.P. 5′-GAAGATGGTGATGGGATTTC-3′	R.P. 5′-AGGGCTGCATTGCAAATATC-3′
MMP-9	F.P. 5′-TGGGCTACGTGACCTATGACAT 3′	F.P. 5′-GTATGGTCGTGGCTCTAAGC-3′
	R.P. 5′-GCCCAGCCACCTCCACTCCTC-3′	R.P. 5′-AAAACCCTCTTGGTCTGGGG-3′
GAPDH	F.P. 5′-ATTGCCCTCAACGACCACTT-3′	F.P. 5′-ACCACAGTCCATGCCATAC-3′
	R.P. 5′-TTACTCCTTGGAGGCCATGT-3′	R.P. 5′-CACCACCCTTGTTGCTGTAGCC-3′
TGF-βR1	F.P. 5′-TGGGACCCACTTCCATTTCCTTCA-3′	
	R.P. 5′-TCCCAAGCCTCATCTGCTCAATCT-3′	
TGF-βR2	F.P. 5′-TGTTGAGTC CTTCAAGCAGACCGA-3′	
	R.P. 5′-ACTTCTCCCACTGCATTACAGCGA-3′	
bFGF	F.P. 5′-CTTTGGCTGCTACTTGGAGG-3′	
	R.P. 5′-GAAGCTTTCCAGCAAAGTGG-3′	
VEGF	F.P. 5′-AGGAGGGCAGAATCATCACG-3′	
	R.P. 5′-CAAGGCCACGGGGATTTTCT-3′	
SDF-1	F.P. 5′-ATGAACGCCAAGGTCGTGGTC-3′	
	R.P. 5′-CTTGTTTAAAGCTTTCTCAGGTACT-3′	

### Co-culture Assays

Normal fibroblasts (NFs) and cancer-associated fibroblasts (CAFs) were obtained from Dr. Andra Frost, Department of Pathology, The University of Alabama at Birmingham (UAB) and from the UAB Tissue Procurement Facility, respectively. The NFs and CAFs were cultured in a Boyden chamber (transwell) system with either parental MDA-MB-435 cells or TGFβ1-silenced clones (MDA-MB-435 TGFβsi). Cells were allowed to grow for 72 hrs and then harvested for RNA isolation. RT-PCR and immunofluorescence were used to determine the effects of TGF-β1 silencing on tumor-stroma interactions.

### Immunocytochemistry

Immunocytochemistry was performed using polyclonal antibodies for PARP, ki67, alpha-SMA, and vimentin in a working dilution of 1:500 (Abcam Cambridge, MA). The slides were then stained with either donkey anti-rabbit or donkey anti-mouse horseradish peroxidase-linked secondary antibodies (1:500 dilution). The stained slides were examined in a Leica microscope under high power (x40).

### *In vivo* studies

All animal protocols were performed following the guidelines of UAB Institutional Animal Care and Use Committee (UAB-IACUC). MDA-MB-435 tumors were developed in athymic nude mice. Six week-old mice were purchased from Charles River and housed in the Animal Care Facility of the University of Alabama at Birmingham. In the experiment to determine the expression levels of TGFβ1 and MMPs in primary, and at metastatic tumor sites, MDA-MB-435 cells were subcutaneously implanted in cohorts of mice. To facilitate metastasis, when the tumors reached a volume of 100 cu mm, they were surgically explanted. In the experiment to determine the role of CAFs in primary tumor growth, a total of four groups, consisting of 12 mice per group, were included in the study. Unmodified MDA-MB-435 cells or MDA-MB-435TGFβsi cells, either alone or in combination with CAFs, were subcutaneously implanted. Tumors were allowed to grow up to 25 days and primary tumors were measured every alternate day to determine growth kinetics.

### Statistical analysis

Results consisting of three or more groups were analyzed using single factor analysis of variance (ANOVA). Analysis of results containing two groups was performed using the Student’s t test assuming unequal variance. Values of p < 0.05 were considered statistically significant.
